# Resonance Effects in Variable Practice for Handball, Basketball, and Volleyball Skills: A Study on Contextual Interference and Differential Learning

**DOI:** 10.3390/sports12010005

**Published:** 2023-12-22

**Authors:** Julius Baba Apidogo, Achraf Ammar, Atef Salem, Johannes Burdack, Wolfgang Immanuel Schöllhorn

**Affiliations:** 1 Faculty of Education and Communication Science, Akenteng Appiah-Menkah University of Skills Training and Entrepreneurial Development, Kumasi P.O. Box 1277, Ghana; japidogo@gmail.com; 2 Institute for Sport Science, Johannes Gutenberg-University Mainz, 55128 Mainz, Germany; acammar@uni-mainz.de (A.A.); burdack@uni-mainz.de (J.B.); 3 High Institute of Sport and Physical Education of Sfax, University of Sfax, Sfax 3000, Tunisia; 4 Research Laboratory, Molecular Bases of Human Pathology, LR19ES13, Faculty of Medicine, University of Sfax, Sfax 3000, Tunisia; 5 Interdisciplinary Laboratory in Neurosciences, Physiology and Psychology, Physical Activity, Health and Learning (LINP2), UPL, Paris Nanterre University, UFR STAPS, F-92000 Nanterre, France

**Keywords:** adaptive stochastic resonance, basketball free-throw, handball shooting, volleyball underarm pass, contextual interference, differential learning, free-play, skill similarity, movement topology

## Abstract

Effective sports training should be attuned to the athlete’s specific conditionings and characteristics. In motor learning research, two often neglected factors that influence this resonance are the learner’s athletic background and the structural diversity of exercises (e.g., relative similarity). In the setting of real-word training with higher external validity, this study examines the effects of three learning approaches (i.e., contextual interference (CI), differential learning (DL), and free-play control condition (CO)) on the parallel learning of handball (HB), volleyball (VB), and basketball (BB) skills, considering participants’ prior sport backgrounds. Forty-five males (15 HB, 15 VB, and 15 BB players) with a mean age of 22 ± 1.4 years and at least 6 years of experience in the mastered discipline voluntarily participated in this study. A pre–post–retention test design including a 6-week-intervention program was employed. During the intervention period, participants engaged in three training sessions a week, with each one lasting approximately 80 min. Each of the three test sessions involved the execution of ten attempts of BB free-throw shooting, HB three-step goal throwing, and VB underarm passing following a blocked order. In terms of short-term (pre–post) gain, only the DL group significantly improved their performance in both non-mastered disciplines (*p* = 0.03, ES = 1.58 for the BB free-throw and *p* = 0.05, ES = 0.9 for the HB shooting tests), with a trend (ES = 0.53) towards an improvement in the performance of the mastered VB underarm-pass skill. In terms of relatively permanent gains, the CI group significantly improved their performances from pre- to retention test only in the non-mastered BB free-throw skill (*p* = 0.018, ES = 1.17). In contrast, the DL group significantly improved their performance at retention compared to the pre-test in both non-mastered BB (*p* = 0.004, ES = 1.65) and HB (*p* = 0.003, ES = 2.15) skills, with a trend (ES = 0.4) towards improvement in the mastered VB test. In both the short-term and relatively long-term, higher composite score gains were observed in DL compared to CI (*p* = 0.006, ES = 1.11 and 0.049, ES = 1.01) and CO (*p* = 0.001, ES = 1.73 and <0.0001, ES = 2.67). In conclusion, the present findings provide additional support for the potential advantages of the DL model over those of CI. These findings can serve as the basis for tailored training and intervention strategies and provide a new perspective for addressing various issues related to individual and situational learning.

## 1. Introduction

For a considerable period, numerous motor learning publications have introduced the notion that the advantages of variable practice are generally accepted [1,2,3]. However, it is noteworthy that the concept of variable practice remains subject to considerable ambiguity, encompassing a spectrum of interpretations related to various dimensions, including variables such as size, type, spatial configuration, and temporal aspects. Importantly, this conceptual ambiguity has resulted in a lack of sufficient scientific evidence, particularly within the context of sports [4,5,6,7,8,9,10], relying primarily on numerous inadmissible generalizations [6,11,12]. 

After extensive experience in sports practice with most versatile movement variations in the context of methodical exercise and game series for the acquisition of complex gymnastic movements [13] and various team games [14], the models of Variability of Practice (VP) [15,16] and Context Interference Learning (CI) [17,18] in particular initiated and inspired a shift in focus from variation for guided acquisition by means of material aids [19,20,21] to the variation of the movement itself for stabilization [8]. However, both approaches insisted on the orientation towards a role model. This role model was intended to be stabilized in the form of movement invariants (e.g., relative forces, relative timing), through the imitation and repetition of these invariants combined with variable parameters (e.g., absolute forces. absolute timing). Although both VP and CI models have consistently demonstrated systematic effects exclusively for movements with few degrees of freedom (DGF) and a predominant reliance on visual input [8,9,11], a relatively unnoticed issue has emerged when attempting to integrate the VP model as a subset of the CI model. Whereas the VP model was originally designed for the stabilization of a single, already automated skill [15], achievable through an arbitrary set of variable parameters, the subsequent CI model served not only to stabilize single skills through the randomized ordering of variable parameters but also to simultaneously acquire different skills with varying relative timing [11].

In both scenarios, these theories provided no insights into the relative distances of the variable parameters or the distribution and relative similarities among the invariants or skills. Should one inappropriately transfer these theories to skills in sports [11], the distinction between, for instance, learning three skills in volleyball or three skills from three distinct sports concurrently would become negligible. Individual findings highlight the influence of movement similarity on learning behavior in movements with few DGF [22,23]. In one instance, this pertains to movements with different topologies [22], while in another instance, it applies to movements sharing the same topology but differing in relative timing and, hence, in their generalized motor programs (GMPs) [23].

For movements with few DGF, this led to the hypothesis that parallel practice of movements with little similarity or different GMPs may result in greater learning effects in the retention test [23]. However, this hypothesis does not provide specific details regarding the nature of these similarities, and it neglects any possible influence of movement topology. In an attempt to overcome this issue, the approach suggested by [3] enumerates different topologies of tennis strokes at a nominal scale level. However, the assessment of their relative similarity remains ambiguous, thereby causing the absolute count to depend on subjective judgements by experts. This issue becomes more pronounced when considering that the CI model does not allow deviations from the prescribed movements [24]. The problematic nature of this endeavor becomes evident when considering recent findings from the field of pattern recognition on the non-repeatability of everyday and sports movements and the inherent noise present across all categories of observation [8,25,26,27,28,29,30]. From a system dynamics perspective, where noise is considered to have a critical influence on the dynamics of phase transitions, such as during the learning process [31,32], the results could alternatively be interpreted as varying variable parameters inducing noise in the system that are insufficient to trigger a phase transition. In contrast, different GMPs do so due to their inherently greater noise [33]. In addition to the challenge of quantifying the similarity of sport exercises, there remains an unresolved gap in sports-related CI research that pertains to the effects of different groups of movement topologies or movement metrics on learning progress [33,34]. 

In sports, the first indications for different influences of exercise combinations could be seen in CI-related volleyball studies with three volley skills [34,35,36,37]. Notably, when practicing the overhead serve and overhead pass concurrently with an underarm pass, the observed learning gains tended to manifest in the upper movement range, suggesting a stronger effect due to the two skills played above the shoulder. Whereas when the underarm serve and pass were practiced together with the overhead pass, greater learning gains were observed in the lower movement range, indicating a larger learning effect due to both underarm skills. Intriguingly, in both scenarios, the two skills occupying similar movement spaces, either above or below the shoulder, appeared to mutually enhance learning due to their increased movement noise.

The two phenomena associated with CI [18,19], namely reduced acquisition and increased learning in random and serial practice compared to blocked learning, are commonly explained by means of memory processes that are associated either with increased elaboration [24], reconstruction [38], or forgetting [39]. While the first model assumes an expansion of the exploration space through additional tasks, the second model sees the conditions more in constant rescheduling, which is, therefore, often associated with the third model of accelerated forgetting through interspersed exercises. However, it is important to distinguish between these latest models, as they differ in their levels of distraction. All three models suggest an overload of the capacity of working memory during the acquisition phase as being responsible for the decreased change in performance. Interestingly, the working memory model they rely on was originally developed only for sequential, visual–spatial content [40] an area where the model now exhibits its highest reliability. However, a comprehensive explanation of how this decreased acquisition rate leads to a subsequently larger increase is still required. Findings from MRI studies provided evidence for corroborating different brain activations during blocked vs. randomized practice [41,42]. However, this has only been observed in movements with few DGF due to constraints associated with the available measurement devices. In contrast to VP and CI, the DL model has always placed a central emphasis on the similarity of exercises, as implied by its name, since similarity can be considered the opposite of difference, and both fall within the family of proximity measures. Pattern recognition methods based on machine learning (ML) that were developed in parallel with DL provide acknowledged and appropriate tools for quantifying similarities of movements across different topological scales [30,40]. These ML methods have proven capable of not only classifying skills with different topologies such as shot put, discus, and javelin throwing [43,44], or activities like running, walking, and handwriting [45], but also skills individually and situated differentiated by emotions [46], fatigue [47,48], music heard [46], or even by pure temporal changes [27,49]. Interestingly, by decomposing what was previously considered movement noise into various movement qualities, they could be assigned to different magnitudes (variance) of noise. Although not yet fully established, these findings coarsely show the biggest differences between skills followed by between individuals vs. followed by between situational conditions within an individual, indicating a gradual decrease in noise levels from skills to individual to situational conditions. 

Through the intentional increase of noise, DL also abandoned the idea of a narrow person- and time-independent prototype [25,34,50] and gave noise an active and constructive influence according to the system dynamics of phase transitions [31,51,52]. With the amplification of fluctuations and without giving augmented feedback, DL in its most extreme form initiated a real self-organizing process as no specific information about a possible solution is provided, not even in terms of guidance towards an intended target behavior [53]. The question of whether the increase in fluctuations was initiated by external or internal factors was, therefore, of secondary importance and could rather be assigned to the fields of pedagogy and didactics. Hence, available literature proposes a framework that not only unifies previous approaches as different forms of noise [26,54] but in parallel suggests a quantitative methodology to address the unresolved issue in other learning models using a holistic ML approach [34,43,54,55,56]. In the context of learning a single movement, several studies have provided evidence supporting the superiority of stochastic DL training over repetitions-based training in the context of skill-related learning [34,56,57,58,59,60,61] as well as equality in strength training [62,63,64]. Meanwhile, successful applications of DL have been broadened with the learning of multiple skills simultaneously, hence reinforcing the superiority of DL over repetitive and CI learning in football [60] and volleyball [34,61,65].

In addition to the initiation of real self-organization, the attunement of the external and internal adaptation of fluctuations with the stochastic resonance principle became an essential feature of DL [66]. Stochastic resonance, derived from physics and employed by differential learning (DL), is achieved within a time delayed feedback loop when a system is exposed to an external force or oscillation that adjusts to the inherent natural frequency time shifted [67]. Given the various experiences and constantly changing boundary conditions on the part of the learner (emotions, fatigue, chronobiology, aging, etc.), the resonance principle includes a corresponding adjustment of the external force to achieve resonance, which is termed adaptive stochastic resonance [68].

This assumes a harmonization of the exercise variations offered by the trainer with the responses exhibited by the athlete, both of which can be understood as stochastic signals [69]. It is only when these elements achieve optimal resonance with each other that the learner can attain a maximum response in the form of maximum learning progress. According to this resonance principle, the role of a coach can be defined as that of a facilitator responsible for maintaining an optimal level of noise in the system. If the magnitude of the athletes’ noise is insufficient, it must be augmented. Since the athletes’ responses to the interventions are generally quite individual [25,30,56], and that such individuality is strongly associated, aside from the emotional and situated influences, with the athletes’ respective experiences, it is important to consider the resonance between the exercises and the athletes’ experiences to optimize the learning rate. Therefore, it is necessary to carry out a double tuning of the exercises. In addition to the inherent similarities among the exercises themselves, it is important to establish harmony and resonance between the exercises and their respective predecessors.

In contrast to CI, research on DL demonstrates distinct advantages over repetitive or blocked learning models, already during the acquisition phase. According to the CI hypothesis, such advantages are typically observed during the subsequent learning phase (i.e., retention). Previous EEG-based brain studies compared the electrical activation of the brain immediately after repetitive CI, and various forms of DL training in badminton. The main findings indicate increased gamma frequencies in the frontal lobe, associated with aspects of working memory after CI, but a shift towards lower frequencies in this brain region following all forms of DL [70,71], which suggests increased stress following the CI model.

Because the variety of movements in the DL sessions during the acquisition phase significantly exceeds the variety in the CI, the benefits in the acquisition phase contradict the cognitive overload theory in the CI model. These inconsistencies suggest a shifting of focus towards the influence of factors such as the topology of movements (degrees of freedom, parallel or sequential, amount of visual control) and similarity of the exercises. Alternatively, it may be beneficial to evaluate the role of expectations and their impact [72]. Such an approach would activate the same neural region associated with working memory, going beyond simply considering the number of exercises.

The aim of this study is to identify potential resonance conditions between prior sporting experiences and learning approaches in the setting of real-word training with higher external validity. For this purpose, we examined the effects of two learning approaches on the parallel learning of three skills, each with a different movement topology, depending on participants’ prior sport background. The three sports that were chosen and in which each group of participants had already achieved an advanced level of proficiency were handball (HB), basketball (BB), and volleyball (VB). Notably, two of these sports (HB and BB) share a higher degree of similarity in their fundamental activities (e.g., catching, throwing, dribbling) compared to the third (VB), which exclusively involves volleyball activities. Within this context, one group engages in free-play and serves as a control group, as they maintain their regular sport-specific practice in a playful manner and exclusively interact with the two other sports’ skills during testing sessions. 

As a fundamental element of scientific research, different predictions are to be derived from the underlying conditions of the theories [73]. In the case of the CI model, which exclusively contains statements relative to blocked learning regarding the two characteristic phenomena and does not vary initial skill levels, its predictive scope remains limited. However, cognitive–psychological explanations regarding the interference phenomenon, primarily attributed to overloaded working memory, suggest that we should anticipate relatively modest improvements in newly acquired skills during the acquisition phase among the DL group compared to the CI. This expectation arises from the greater diversity of exercises encountered in the DL approach. 

In contrast, the DL theory proposes that individuals with advanced proficiency in the tested sport would (i) exhibit superior baseline, due to the resonance between prior experience and the test skill, and (ii) experience smaller increases in performance during and after the intervention than advanced learners from other sports, due to their relatively reduced noise in their movements. Additionally, because of the increased noise in the DL model, a continuous increase in performance would be observed in all tested skills among the DL group. Furthermore, it can be hypothesized that the DL group would exhibit greater improvement in the less-familiar skills as compared to the CI and control groups [34], due to the lower noise levels per skill in these two groups [74]. Given the familiarity of the control group with the noise levels often encountered during their regular training sessions and the absence of any change in noise intensity during the intervention, an absence of significant performance improvement would be hypothesized in this group.

## 2. Materials and Methods

### 2.1. Participants

The sample size was calculated a priori based on procedures suggested by Beck et al. [74] and using the software G∗power (version 3) [75]. F-test and ANOVA were set as a test family and statistical test, respectively. Values were set at 0.05 for α and 0.95 for power. The effect size was estimated to be 0.5 (medium effect). The required sample size for this study was 45. The participant recruitment process started on 5 January 2023, for a period of six weeks. Recruitment efforts targeted local basketball, handball, and volleyball clubs through a multifaceted approach involving flyers, posters, and direct communication with coaches. A total of fifty potential participants underwent screening for eligibility criteria. Forty-five male participants (15 handball players, 15 volleyball players, and 15 Basketball players) with a mean age of 22 ± 1.4 years old and with at least 6 years of experience (i.e., 3–5 training sessions per week) in the mastered discipline were successfully recruited to voluntarily participate in this study.

**Figure 1:**
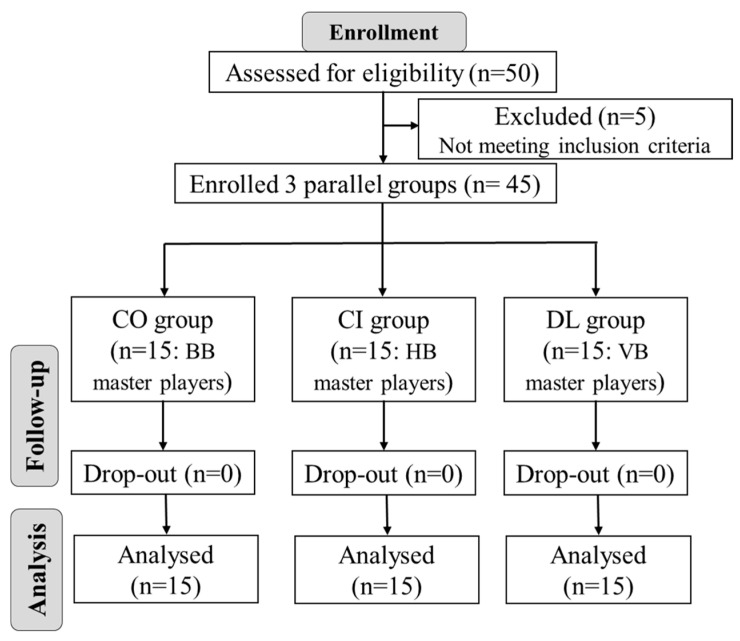
CONSORT flow diagram.

None of the 45 participants had prior experience in the other games; thus, they were either only volleyball players, only basketball players, or only handball players. The handball players formed the contextual interference (CI) group, the volleyball players formed the differential learning (DL) group, and the basketball players formed the control (CO) group. To give a clearer indication of whether or not practicing the three disciplines simultaneously improved the non-familiar disciplines, the groups were assigned based on their familiarity with each discipline. Notably, the study experienced no participant dropouts, as illustrated in Figure 1. All participants provided written consent to participate in the study following a comprehensive explanation of the study’s objectives. The procedures adhered to the principles outlined in the Helsinki Declaration guidelines and received approval from the institutional review committee. The study was conducted according to the Declaration of Helsinki and was also approved by the institutional review committee of Akenten Appiah-Menkah University of Skills Training and Entrepreneurial Development (AAMUSTED/K/RO/L. I/322).

### 2.2. Experimental Design

**Figure 2:**
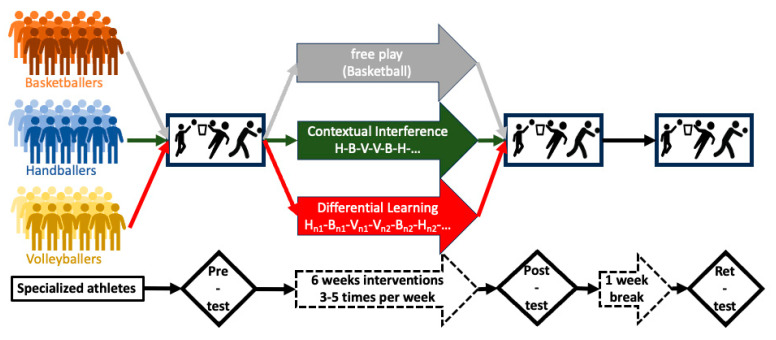
Study design. (B: basketball-test/exercise; H: handball-test/exercise; V: volleyball-test/exercise; H_ni_, B_ni_, V_ni_: variants of H, B, V).

A pre–post–retention between-subject test design was employed for the study (Figure 2). After the pre-test, the intervention started and lasted for 6 weeks. Participants engaged in training sessions thrice a week (Mondays, Wednesdays, and Fridays), each training session lasted approximately one hour and twenty minutes. Following the final intervention training session, an immediate post-test evaluation was administered, with a retention test conducted one week thereafter. Prior to engaging in the intervention activities, a customary warm-up period of 10 min was undertaken. All training and test sessions were performed at the same time of day (in the afternoon), as previously suggested by Gebkenjans et al. [76] to minimize the effect of diurnal biological variations [77]. The training and testing sessions were carried out in the same open field. All players reported having normal sleep nights with no sleep deprivation or poor sleep quality prior to all test sessions. The study evaluated three distinct skills originating from the three sporting disciplines, characterized by varying degrees of kinematic similarity. Specifically, the skills encompassed basketball free-throw shooting, handball three-step goal throwing, and volleyball underarm passing. Adopting a counterbalancing procedure, each of the three test sessions involved the execution of ten attempts per skill, following a blocked order. 

### 2.3. Test Design

**Figure 3:**
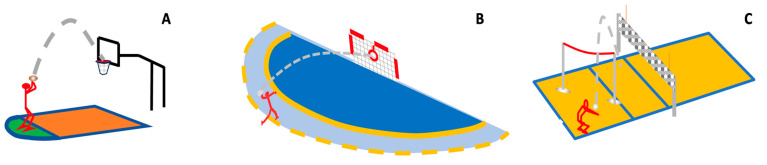
Schematic representation of the three tested skills, (**A**) basketball free-throw shooting test; (**B**) 3-step handball shooting test; (**C**) volleyball underarm pass test.

The schematic representation of the study’s tested skills is presented in Figure 3. Three skills from different sports were used, first in contrast to the previous study [34], and second, because the noise caused by various skills is the biggest in comparison to the individual or situated noise. The basketball skill was carried out in accordance with the basketball free-throw shooting test. The handball skill aligned with the handball shooting test as guided by [78,79] and the volleyball underarm pass adhered to the guidelines established in [80], the volleyball skill test manual [80]. Each of these distinct tests corresponded directly to a fundamental skill within their respective sporting disciplines. 

#### 2.3.1. Test 1 A: Basketball Free-Throw 

This test aimed to assess the accuracy of basketball free-throw shooting skill [81]. Participants occupied the free-throw line, receiving balls from a designated feeder positioned in front of the player at a distance of at least 9 m. Shots were directed towards the regulation basketball rim, measuring 18 inches (48.75 cm) in diameter. The free-throw line was situated at 4.6 m from the backboard, and the rim was positioned at a height of 10 feet (approximately 3.05 m) from the floor on a standard basketball court. Successful entry into the rim garnered 2 points, while contact with the rim without subsequent entry earned 1 point. 

#### 2.3.2. Test 1 B: Handball Shooting

This test sought to measure shooting accuracy in handball [78]. Participants positioned themselves behind a 9 m line and received a pass from a designated ball feeder. Executing three steps, initiated with the leg opposite the throwing arm, participants were required to release the ball using a single hand without bouncing and jumping. The target was a handball post measuring 2 m by 3 m, featuring a centrally positioned circular object measuring 1 m in diameter. Points were awarded based on hitting the target (2 points), passing through the goal post without target contact (1 point), and non-scoring instances when off the goal post.

#### 2.3.3. Test 1 C: Volleyball Underarm Pass

This test targeted the accuracy of the underarm volley pass. They received throws from participants positioned on the opposite court and were required to pass the ball over a rope elevated to a height of 2.46 m. The objective was to direct the ball into a 3 m × 4 m target area located on the participant’s court. Successful targeting of the area led to the allocation of 2 points, while contact with any part of the marked lines within the target area resulted in 1 point [80,81,82] (AAHPER).

Each participant executed 10 attempts for each of the three tests, rendering a potential score range of 0 to 20 points for each subtest. 

### 2.4. Training-Interventions

The intervention lasted for six weeks, during which participants engaged in training sessions three times a week. Following a ten-minute warm-up comprising jogging and minor games (unsimilar to the tested skills), participants transitioned into their designated groups for each scheduled practice session. The CI group (advanced handballers) adopted the CI model. The C1 group’s training regimen revolved around executing the three skills randomly. No skill was consecutively repeated more than twice, thereby generating a pattern of execution from handball shooting (H) to basketball shooting (B) to volleyball underarm pass (V) (e.g., H-B-V-B-V-B-H-V-H, etc.). These executions were performed iteratively, with ongoing corrections and augmented feedback provided by an experienced coach following each training trial. 

The DL group (advanced volleyballers) adhered to the same sequence as the CI group but incorporated the DL model. The DL approach entailed adding perturbations, or amplified “noise,” to the specific exercises. Accordingly, a sequential execution pattern was established as Hn1-Bn1-Vn1-Bn2-Vn2-Bn2-Hn3-Vn3-Hn3 and so forth, ensuring no repetition of skill sequences or actions. Notably, no corrective feedback was administered, and the emphasis lay in intensifying perturbations across the three skills spanning the sporting disciplines. 

In both the CI and DL groups, each participant undertook 10 trials per session for each skill. Throughout the entirety of the training interventions, both the CI and DL groups accumulated 570 relevant ball contacts (10 trials × 3 skills/session × 3 sessions/week × 6 weeks). Throughout the intervention period, six trained ball passers facilitated the provision of balls for participants to execute the skills. The CG group (advanced basketballers), in contrast, was not exposed to alternate activities or practices after the warm-up period. Their engagement was limited to a standard 5-on-5 basketball game lasting 43 min, encompassing the entire court area, which concluded the session. Figure 2 summarizes the study design and the intervention programs.

### 2.5. Data Analysis

A comparative analysis was conducted among the three groups, focusing on their performance outcomes across individual skills and combined non-similar movement skills (i.e., composite score). To ascertain the composite score, the mean values derived from the three individual skills—BB free-throw, HB three-step shooting, and VB underarm pass—across pre-, post-, and retention tests were employed (e.g., composite score at pre-test = mean of BB, HB, and VB scores at pre-test). The retest reliability, or internal consistency, of the respective skill tests was examined within a subset of the groups. Specifically, three participants from each group undertook the relevant tests within a one-week interval. The determination of internal consistency involved the calculation of utilizing data from both the initial and subsequent weeks; for the purpose of evaluation, a Cronbach alpha was >0.8.

Descriptive statistics were reported as the mean ± standard deviation (SD). The normality of the data was assessed using the Shapiro–Wilk test. For comparing differences between groups (DL, CI, and CG) and time slots (pre-test, post-test, and retention test), a two-way analysis of variance (ANOVA) was conducted, where parametric assumptions were met, followed by post hoc pairwise comparisons using the Bonferroni adjustment. When the data did not meet parametric assumptions, the F1-LD-F1 model was utilized. This model generates an ANOVA-type test statistic (ATS) to assess the main effect of group, time, and the interaction group × time. ATS were reported due to their suitability for smaller samples, and modified ATSs (ATS_Mod_) with Box approximation were used for the between-subject factor of the intervention group. Subsequently, Dunn’s test was employed for between-group comparisons, and the Wilcoxon test was applied for comparisons between different time intervals. Both post hoc tests were adjusted using the Bonferroni method.

To ascertain the significance of differences in the delta change between various testing times and the composite scores across the three tests, the appropriate one-way ANOVA or Kruskal–Wallis test was performed based on parametric or non-parametric considerations, respectively. This was followed by post hoc pairwise comparisons or Dunn’s test, both adjusted with the Bonferroni correction. The delta change (Δ) was calculated as follows: Δ pre–post = score at post-test − score at pre-test; Δ post–retention = score at retention test − score at post-test; Δ pre–retention = score at retention test − score at pre-test.

Effect size statistics (ηp^2^ and η^2^[h]) were computed to evaluate the magnitude of differences, classified as small (0.01), moderate (0.06), and large (0.14) according to Cohen [83]. Statistical significance was established a priori at *p* < 0.05 for all analyses. The statistical procedures were implemented using the R programming language [84].

For ANOVAs and post hoc tests involving normally distributed data, the “afex” package [85] and “emmeans” package [86] were employed, respectively. The Kruskal–Wallis, Dunn’s, and Wilcoxon tests were conducted using the “rstatix” package [87]. The F1-D1-F1 model was executed using the “nparLD” package [88].

## 3. Results

### 3.1. Comparative Analysis across Single Skills

**Figure 4:**
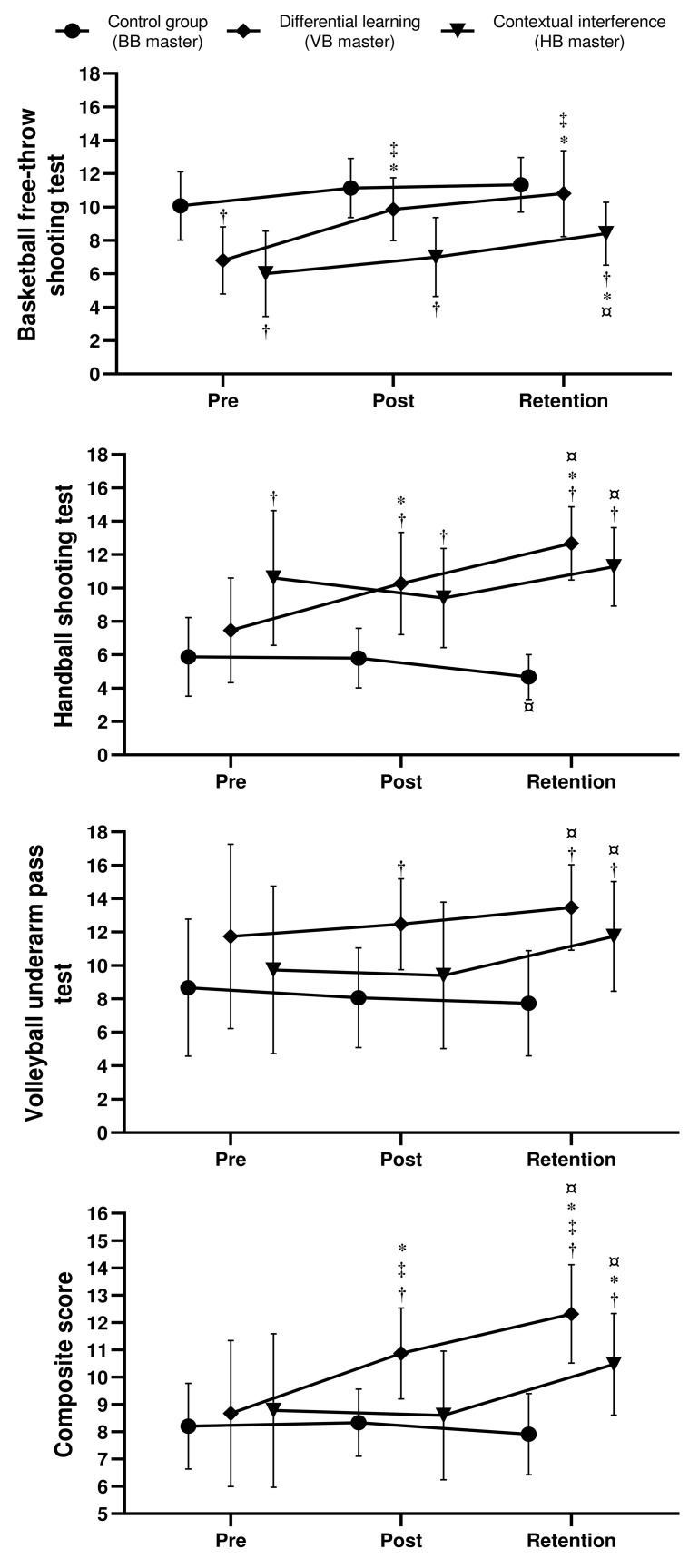
Mean ± SD of the basketball free-throw, handball shooting, the volleyball underarm pass tests, and the composite score for control, contextual interference, and differential learning groups at pre-, post-, and retention test timepoints. *: significantly different compared to pre; ¤: significantly different compared to post; †: significantly different compared to the control group; ‡: significantly different compared to contextual interference.

The results of the BB free-throw, HB shooting, and the VB underarm pass tests for the different groups at pre, post, and retention tests are presented in Figure 4. 

The F1.LD.F1 model for the BB free-throw test points revealed significant effects of group (ATS_Mod_ (1.98, 41.32) = 20.58; *p* < 0.001; ηp^2^ = 0.49), time (ATS (1.86, ∞) = 28.76; *p* < 0.001; ηp^2^ = 0.41), and a significant interaction of group × time (ATS (3.16, ∞) = 3.32; *p* = 0.017; ηp^2^ = 0.14). The pairwise comparison showed that scores significantly increased for DL group at post-test (*p* = 0.003) and retention test (*p* = 0.004) compared to pre-test. However, scores for CI significantly increased at retention test compared to pre- (*p* = 0.0179) and post-tests (*p* = 0.0079). At pre-test, CG reported higher scores compared to DL (*p* = 0.0039) and CI groups (*p* = 0.0003). The CI group reported lower scores compared to the CG and DL groups at post-test (*p* < 0.0001 and *p* = 0.0203, respectively) and retention test (*p* = 0.0012 and *p* = 0.0168, respectively).

Regarding the HB shooting test, the F1.LD.F1 model showed significant effects of group (ATS_Mod_ (1.93, 39.2) = 23.92; *p* < 0.001; ηp^2^ = 0.53), time (ATS (1.59, ∞) = 8.22; *p* < 0.001; ηp^2^ = 0.16), and a significant interaction of group × time (ATS (2.84, ∞) = 14.58; *p* < 0.001; ηp^2^ = 0.41). The shooting score for DL group significantly increased from pre-test to post- (*p* = 0.05) and retention test (*p* = 0.003), as well as from post- to retention test (*p* = 0.027). For the CI group scores significantly increased only from post- to retention test (*p* = 0.004), while they decreased for the CG (*p* = 0.016) during the same time. The shooting score for CI group was higher compared to CG at pre- (*p* = 0.0033), post- (*p* = 0.0028), and retention tests (*p* < 0.0001). Scores for the DL group were higher compared to CG at post (*p* = 0.0003) and retention tests (*p* < 0.0001). 

Since the baseline for BB was not equal between the CG and the two other groups, any revealed difference between CG and CI and CG and DL at post-test and/or retention test should be interpreted with caution. The same applies to the difference between CG and CI in the handball shooting test. 

The two-way ANOVA for VB underarm pass scores revealed a significant effect of group (F (2, 42) = 5.79; *p* = 0.006; ηp^2^ = 0.22), time (F (2, 84) = 3.74; *p* = 0.028; ηp^2^ = 0.08), and a significant interaction group × time (F (2, 84) = 3.27; *p* = 0.015; ηp^2^ = 0.14). The underarm pass scores significantly increased at retention test compared to post-test for both CI (*p* < 0.0001) and DL (*p* = 0.0333). The DL group reported higher scores compared to CG at post-test (*p* = 0.0101). Also, CG score was lower at retention test compared to CI (*p* = 0.0068) and DL (*p* < 0.0001). 

### 3.2. Comparative Analysis across the Composite Score

Regarding the composite score, the F1.LD.F1 model showed significant effects of group (ATS_Mod_ (1.95, 39.88) = 7.92; *p* = 0.001; ηp^2^ = 0.27), time (ATS (1.53, ∞) = 25.02; *p* < 0.001; ηp^2^ = 0.38), and a significant interaction of group × time (ATS (2.73, ∞) = 15.77; *p* < 0.001; ηp^2^ = 0.43). The composite score for the DL group significantly increased from pre-test to post- (*p* = 0.008) and retention test (*p* = 0.002), as well as from post- to retention test (*p* = 0.005). For the CI group, scores significantly increased from pre- to retention (*p* = 0.034) and from post- to retention test (*p* = 0.002). The composite score for the DL group was higher compared to CG at post- (*p* = 0.0005) and retention tests (*p* < 0.0001), and higher compared to CI at post- (*p* = 0.006) and retention tests (*p* = 0.049). Scores for the CI group were higher compared to CG at retention tests (*p* = 0.003).

### 3.3. Comparative Analysis across the Short-Term and Relatively Permanent Gains

**Figure 5:**
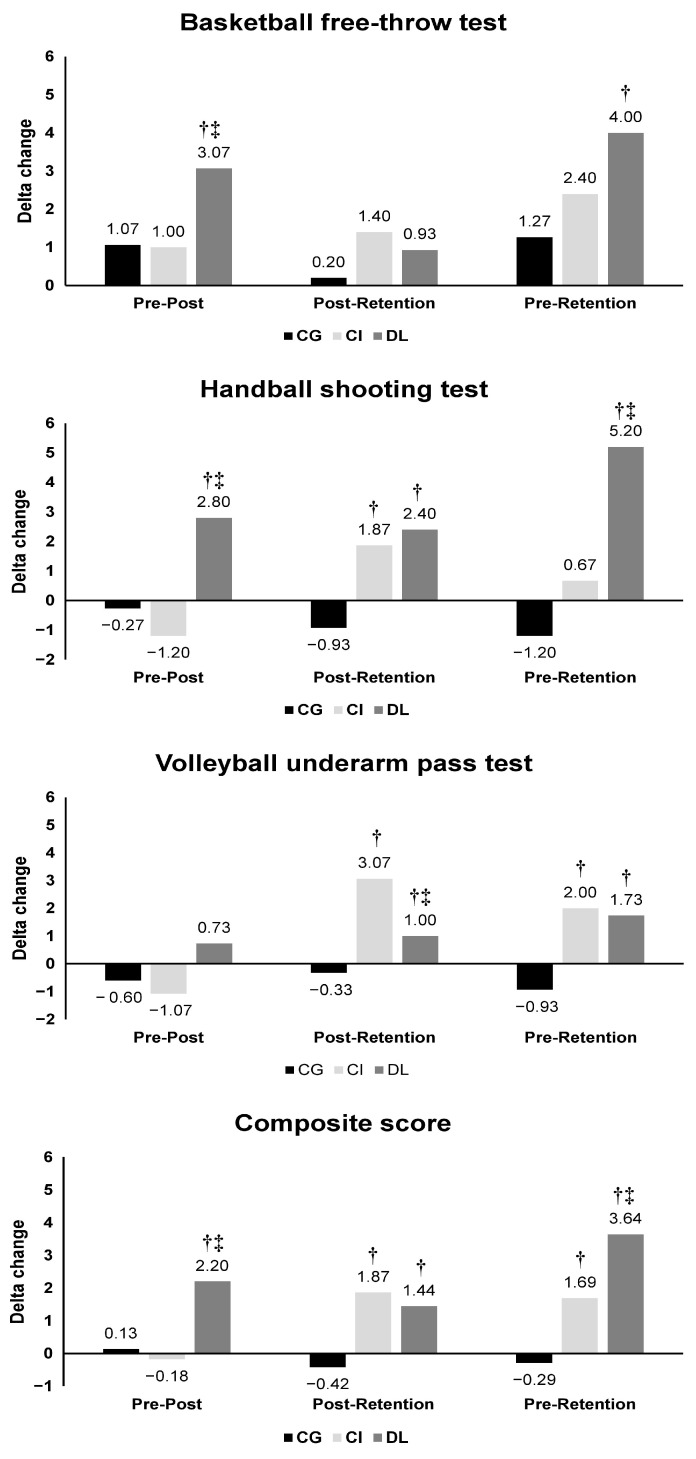
Mean ± SD of the different delta change (Δ) for the basketball free-throw, handball shooting, and the volleyball underarm pass performances. †: significantly different compared to the control group; ‡: significantly different compared to contextual interference.

To overcome the differences found at baseline between the CG and the other groups, particularly in the BB and HB performance outcomes, one-way ANOVA analyses were performed to detect group effects in the delta change (Δ) between the different testing timeslots (i.e., Δ pre–post, Δ pre–retention, and Δ post–retention). The results are presented in Figure 5. A summary of the short-term (Δ pre–post) and relatively permanent (Δ pre–retention and Δ post–retention) gains are also summarized in the figure abstract.

#### 3.3.1. Group Effect on the Short-Term Gain (Δ Pre–Post) 

Significant group effects were found in Δ pre–post for the BB free-throw test (F (2, 42) = 3.95, *p* = 0.027, ηp^2^ = 0.17) and the HB shooting test (F (2, 42) = 8.12, *p* = 0.001, ηp^2^ = 0.28) with a higher change in DL compared to CG and CI for the BB free-throw test (*p* = 0.003 and 0.017, respectively) and HB shooting test (*p* = 0.0208 and 0.004, respectively). There was a non-significant group effect for the VB underarm pass test in Δ pre–post (H (2) = 2.31, *p* = 0.315, η2[h] = 0.01). Regarding the composite score, a significant group effect was found in Δ pre–post (H (2) = 14.7, *p* < 0.001, η^2^[h] = 0.3) with a higher change in the DL group compared to CG and CI (*p* = 0.0008 and 0.001, respectively).

#### 3.3.2. Group Effect on the Relatively Permanent Gain (Δ Post–Retention and Δ Pre–Retention) 

Δ post-retention:

Significant group effects were reported in Δ post–retention for the HB shooting test (H (2) = 20.9, *p* < 0.001, η^2^[h] = 0.45) and the VB underarm pass test (F (2, 42) = 27.9, *p* < 0.001, ηp2 = 0.57) with lower changes for CG compared to CI (*p* < 0.0001 for both HB and VB tests) and DL (*p* < 0.0001 and *p* = 0.006, respectively, for the HB and VB tests) and a higher change for CI compared to DL (*p* < 0.0001) for the VB test. There was a non-significant group effect for the BB free-throw test in Δ retention–post (H (2) = 4.2, *p* = 0.123, η^2^[h] = 0.05). Concerning the composite score, a significant effect was revealed (H (2) = 24.9, *p* < 0.001, η^2^[h] = 0.055) with higher changes in CI and DL groups compared to CG (*p* < 0.0001 and *p* = 0.0001, respectively).

Δ pre-retention:

Significant group effects were found in Δ pre–retention for the BB free-throw test (F (2, 42) = 4.63, *p* = 0.015, ηp^2^ = 0.181), the HB shooting test (F (2, 42) = 20.18.2, *p* < 0.001, ηp^2^ = 0.49), and the VB underarm pass test (F (2, 42) = 3.54, *p* = 0.038, ηp^2^ = 0.14) with higher Δ in DL compared to the CG group in the BB (*p* = 0.004), HB (*p* < 0.0001), and VB (*p*= 0.034) tests. CI group showed higher Δ compared to CG only in the VB test (*p* = 0.0206). Importantly, a higher change was reported in DL compared to CI in BB free-throw test and HB shooting test with significant differences between groups in HB test (*p* < 0.0001) and a trend toward a significant difference in BB test (*p* = 0.08, ES = 0.6) A significant group effect was found in the composite score (F (2, 42) = 22.9, *p* < 0.001, ηp^2^ = 0.52) with higher changes in DL compared to CG (*p* < 0.0001) and CI groups (*p* = 0.0015), as well as higher changes in CI compared to CG (*p* = 0.0017).

## 4. Discussion

The aim of this study was to identify possible short- and mid-term resonance phenomena between prior experience and the CI and DL approaches in the setting of real-word training conditions dependent on the similarity of exercises used.

First, the results of the pre-tests confirm the expectation that the specialized athletes outperformed the non-specialized ones in the skills of their particular sport. For the HB and BB players, the differences were statistically significant. According to the potential law of neural adaptation [89] and the typical learning curves in the motor domain [90], it could be assumed that the specialized athletes experience the smallest performance gains in their specific skills during the intervention period that correspond to the stabilization of an already acquired skill. The present findings confirm this assumption in the BB and HB players, who followed the free-play (CO) and the CI models, respectively; but surprisingly not for the VB specialists who followed the DL model. From the DL model point of view, the applied noise should increase with the level of advancement to stay in resonance with the changing characteristics of the athlete. It seems that the VB specialists, who practiced according to DL, experienced the corresponding amount of noise that resonated with the athletes’ experience and, therefore, led to performance increases that, impressively, exceeded the VB beginners of the free-play group and were comparable to those practicing according to the CI model. The increase in performance of advanced VB players, which is comparable to the increase in performance of beginners, leads to the question to what extent classical learning curves are largely due to decreasing responsiveness and to what extent this can at least be reduced by DL. In contrast, the continuation of training that the BB specialists were used to or the variances that the HB specialists experienced during CI did not pass the threshold of noise that would allow short-term and/or relatively permanent gains in the mastered skills. This was evidenced by the absence of significant performance progress from baseline to post-test as well as from baseline to retention test in the BB free-throw among the CO group (BB players) as well as in the HB shooting among the CI groups (HB players). From a cybernetic pedagogy point of view, all three groups would have experienced comparable objective information, but the DL approach contained enough continuously adapting, subjective information according to the pre-knowledge to keep the learning rate on a higher level than the CI approach with skills from different sports or the free BB play [11]. 

The BB specialists (CO) did show only slight, non-significant increases in the BB-specific skill in the short- and medium-term after free basketball play, which has the biggest representativeness with the target exercise, but these were within the range of chance (*p* = 0.140). At least at the level of advanced players, this massively calls into question approaches that recommend training games exclusively by playing them [91]. The HB specialists, who experienced CI learning, did show the absolute phenomenon of interference with a slight, non-significant short-term decrease in performance from pre- to post-test and a significant increase after the retention phase in the medium-term. However, when taken together, the CI and the CO only came up with a slight overall increase in the mastered skill performance that did not reach the level of significance. In contrast to this, a continued increase in the mastered skill performance, evidenced by a non-significant increase from pre- to post-test followed by a significant one in the subsequent learning phase, could already be identified in the VB specialists due to increased noise during the DL-based practice process. 

In light of the cognitive–psychological explanatory models for CI learning, the slight interference effects observed in the HB and VB skills among the handball specialists engaged in CI learning could be attributed to an overload of working memory (cognitive load theory). However, this effect does not hold for the non-mastered BB skill within the same group. These results align with the findings of a recent meta-analysis from our team that put into question the generalization of the CI effects in sport practice [9]. Similarly, in accordance with findings from other recent research from our team [34], the cognitive load theory fails to explain the greater short-term performance gains in the VB specialists engaged in DL learning, where a corresponding number of skill variants are performed in addition to the three tested skills. Indeed, according to the CI model and the cognitive load theory, these additional variants of the DL model should have led to an even higher overload of the working memory, which should have been accompanied by even worse performance compared to CI during the post-test. However, the present results showed that the DL group exhibited significantly higher short-term gains in BB- and HB-related skills, as well as a trend towards higher gains in the VB skill (ES = 0.56), which led to a significantly higher pre–post change in the composite score of the three skills. The idea of the necessity of correct movement execution as frequently as possible, as required in CI learning [24], also cannot be reconciled with the findings on DL [66]. In contrast, the DL model assumes benefits in learning movements with many DGFs, not solely through the implementation of added noise but also through the downregulation of frontal brain areas to a lower frequency range [92]. This shift in brain activity may be attributed to factors such as stress reduction [71,92,93]. The extent to which the activation of frontal areas in the higher frequency range [70,71], following CI is attributed to factors such as the attempt to recall all executions [94], heightened disparities between expectation and outcomes [72], social pressure, or past learning experiences remain open questions; further research is needed to elucidate these aspects. In addition to the complex issues tied to the control of frontal brain areas, it is noteworthy that the concept of noisy training in DL aligns with experiences in the training of artificial neural networks (ANN) within the field of ML. In the context of ML, it has been observed that training an ANN with an appropriate degree of noise around the target of learning results in enhanced robustness during subsequent applications [32,50,95]. It is, therefore, important to emphasize that the optimal level of noise depends on how the ANNs have been pretrained. 

From the perspective of the DL model, and according to more recent approaches examining the CI phenomena, it has been observed that random CI learning first induces stress in the learner [94,96] as evidenced by an increased frequency pattern of the frontal brain areas. This stress, in turn, was suggested to inhibit learning behaviors in the motor areas in the short-term [71,93]. To what extent the stress-associated higher brain frequencies are a necessary condition for a subsequent restructuring of the activation potentials requires further research. On the other hand, it can be argued that the multitude of skill variations in DL serves as a mitigating factor against medium- and long-term overload of the frontal lobe, thereby facilitating motor learning of movements with many DGF [9]. Future research should aim to demonstrate the extent to which the lack of augmented feedback or correction contributes to the reduction in stress levels.

Moreover, from the DL model perspective, the CI intervention for HB specialists as well as the free-play intervention for BB specialists would not elicit sufficient noise in the mastered HB or BB skill, crucial to inducing behavior change that persists over time [74]. In contrast, amplifying the noise in DL seems to provide sufficient noise to trigger behavior changes in terms of increased resilience to greater disturbances, even among advanced players, e.g., VB specialists. The provided noise in DL seems to resonate with the athlete’s noise, even at different levels of performance. Even if one desires to emphasize the novelty and unfamiliarity associated with DL, and the potential for increased motivation that arises from this novelty, this exactly aligns with the principles of DL theory, since it involves reinforcing novel stimuli and noise without repetition. The athlete is constantly confronted with novel experiences. The constant confrontation with “novelty” is also in accordance with the theory of subjective information from the field of cybernetic pedagogy [11,97]. According to this theory, when repeating an exercise, the objective information mainly remains the same, but the subjective information, which depends on the learner’s prior knowledge, becomes smaller with each repetition because redundancy increases. To achieve or keep an individual’s maximum learning rate, the objective information should change constantly to keep the subjective information for the learner correspondingly high, i.e., new information must be presented constantly. Correspondingly, the increased noise applied in DL seems to resonate more with the athletes’ noise, regardless of whether the noise stems from previous experiences or from the inherent similarities of the practiced skills. In this context, it would be interesting to see whether the noise levels are dependent on the intro- or extraversion of an athlete. 

The learning processes exhibit variations when learning skills that fall outside the learner’s specific discipline or prior training, which can be considered as acquisition. In this context, the BB group, which primarily engages in BB training through free-play without practicing HB and VB skills, serves as an appropriate control group for studying the acquisition of these two novel skills. While they do not directly practice HB and VB, their active participation in free BB play for an equivalent duration aims to effectively compensate for eventual confounding factors such as muscular, metabolic, or motivational aspects. This control group shows no statistically significant changes in performance on the HB and VB skill tests, neither after intervention nor after retention phases. The consistent slight deteriorations fall within the range of random fluctuations. For the HB specialists following the random CI protocol, the BB and VB skills are the non-mastered and new skills to learn (=acquisition). In this regard, the BB skill, through catching, throwing, and dribbling, is considered more similar to HB compared to the VB skills. Contrary to expectations derived from the CI model, the HB specialists showed increases in the BB free-throw performances in both the acquisition and retention phases, with significant *p*-values in the retention one. In contrast, with respect to the VB skill, they behaved in accordance with the CI model and showed a slight short-term interference followed by a significant performance enhancement in the subsequent learning phase. Here, the degree of similarities between the mastered discipline or skills and the ones to be acquired seems to influence the learning behavior. The additional noise in the BB intervention caused by random CI practice does not seem to cause too much stress for HB players and thus does not lead to interference during acquisition. From a DL model point of view, the applied noise caused by the transition between different skills seems to surpass a necessary threshold for performance enhancement without being attenuated by stress factors to achieve optimal resonance. In contrast, the VB skill for HB specialists, with its greater dissimilarity, seems to lead rather to stress during the acquisition phase, resulting in excessive noise and thus to a drop in performance. This performance decline appears to trigger a subsequent overcompensation in the retention phase. Notably, only the behavior of the HB group in terms of VB skill can be attributed to the CI model. The behavior of the HB group following the random CI schedule is more likely to be explained by the DL model of stochastic resonance. The noise level associated with the new BB skill falls within the extended noise range of the HB skills, thereby facilitating the learning process and resonates with the noise of the learners. Conversely, the noise generated by the VB skill is considerably away from the catchment area of the HB skills. In this case, it is reasonable to assume that separate training of the network as far as possible is preferable.

Evidence for the influence of prior experience on specific cognitive performance and its transferability was provided in a study conducted by Abernethy et al. [98], where professional team athletes effectively used their already-acquired tactical knowledge to their advantage in other team sports. Similarly, O’Keefe et al. [99] provided evidence for the transfer of motoric performance in skills with high similarities, such as the overhand arm throw and badminton overhead clear. However, both studies primarily focused on specific and general transfer in terms of performance but failed to consider the influence of different initial skill levels on learning, as well as the mutual influence on other skills. While it seems plausible to identify learning transfer between movements that show similar kinematics where comparable muscle activations can be assumed [44], it becomes more challenging to establish such a transfer between two less similar movements, like the HB target throw and the BB free-throw. It would be interesting to explore in future studies potential interaction with a volley skill such as the single-handed overhead serve, that exhibits greater similarity to both HB and BB movements than the double-handed underhand pass. Such an approach could provide further insight to the issue of whether the transfer is more influenced by the volleying activity or the double-handed activity.

Compared to the effects of the CI model, the effects in the DL group were more consistent. The VB specialists showed similar behavior in conjunction with the DL model for both non-mastered HB and BB skills. In both skills, increases in performance are seen in both the acquisition and the retention phases, with statistically significant improvement in most of the cases. Interestingly, in both tested skills, the DL group even reached the level of the specialists in the retention test, as evidenced by the absence of significant differences between the performances of the DL and CO groups in the BB free-throw test, as well as the DL and CI group in the HB shooting test. If we compare the increases in the BB skill performance in the CI and DL groups, which could be considered a non-mastered skill (=acquisition) in both cases without considering the similarity of the sports, the DL group significantly outperforms the CI group in the retention test. Although the BB skill is not part of the usual training activities for either the HB or VB specialists, the skill is mastered by all of them in its rough form from the beginning. More precisely, then, for all athletes, the study has to consider a stabilization process of an already mastered movement that merely began at different levels. However, a different structure (color) or level of noise would be necessary in the case of acquiring more complex movements, such as pole-vaulting or a Tsukahara-vault in gymnastics. 

While previous studies on CI and DL have always focused on the learning of multiple movements within the same sport, the present study investigated movements from different sports that are also understood as larger noise. This provides a different approach to the problem of motor diversity or physical literacy in the context of sports pedagogy and physical education. While increasing noise in single movement repetitions serves to make the athlete’s system more stable against disturbances while repeating this specific movement, increasing noise in the context of motor variety or physical literacy represents a preparation for disturbances of movements in everyday life (coping with stumbling, crossing obstacles, etc.) and largely, thereby, serves health. For this reason, the endorsement of motor diversity has been a long-standing recommendation for the purpose of character development, promoting good health, and preparing individuals both physically and mentally. This recommendation emerged with Gutsmuths [100] in 1804 and was particularly relevant during the emergence of meritocracy in the 18–19th centuries [101,102]. Currently, there is a renewed interest in motor diversity in the Anglo-Saxon-speaking countries. However, it is important to acknowledge that both phenomena share the common principle of enhancing the learner’s preparedness for diverse future scenarios through the introduction of a wider range of exercise elements. Nonetheless, it is worth noting that this occurs on distinct scales of similarity.

Overall, in accordance with the finding of a recent meta-analysis [9] the present study provides further notable limitations to the generalization of the CI model in the sport context. The predictions based on the CI model are confirmed only to a very limited extent, and most of the present results contradict the CI model. In contrast, most predictions based on the DL model experienced corroboration. Based on extensive experience in using ANN for pattern recognition [34,46,56,103,104] in combination with findings from previous DL studies on single (e.g., tennis serve [105], hurdle sprint [106]) and multiple sports (e.g., football [60] and volleyball [65]), and everyday movements [49], three basic principles of ANNs training can be considered to have been transferred to DL: (1) To be effectively trained, an ANN must be trained with noise surrounding the major object of interest. (2) The level of noise should be raised to a threshold where, in the application scenario, interpolation becomes possible instead of extrapolation. (3) The noise must be attuned not only based on the intention (stabilizing existing or acquiring or refining new skills) but also on the prior training of the ANN (e.g., sports experience and individually) [107,108]. The first principle corresponds to learning a skill with stochastic disturbances in DL (e.g., VB serve with varying joint angles). The second one is realized in DL with the amplification of the fluctuations within a skill up to the border of the possible solution space (e.g., VB serves too long or too short, to the left corner, to the right corner, topspin, floating, etc.). The third one corresponds to the suggested adaptive stochastic resonance model [54,107] according to which the (external) noise provided by the coach has to be attuned to the (internal) noise of the athlete, characterized by individual and situational characteristics. While the first two principals have already been extensively and successfully applied in DL, there is currently only sufficient theoretical and plausible evidence for the third principle. From a scientific perspective, the present study represents an initial step in quantitatively investigating this aspect in parallel.

The limitations of the present study are already given by the specific boundary conditions of the investigation. Given the study’s design and the employed statistics [109], the study does not assert generalizability. Based on the original interpretation of Fisher’s statistics [110], the numerous statistically significant results suggest merit to pursuing further research in the proposed direction.

## 5. Conclusions

The present findings provide additional support for the potential advantages of the DL model over those of CI. This is evident in the observed short-term and relatively permanent gains across various performance outcomes in both mastered and non-mastered skills, as well as the higher composite score gains when adopting the DL approach compared to the CI. 

In addition to this, the present study is merely a first step towards approaching the numerous issues related to individual and situational learning from a different perspective. Instead of testing infinite numbers of constraints and their interactions and hoping that the athlete or coach will remember them, or seeking elusive individual key parameters that are subject to temporal variation, the DL approach rather fosters the ability to adapt to many different situations, being aware that complex systems are most sensitive to initial conditions that ask for broader solutions. Rather than simply paying further lip service to “repetition without repetition” devoid of practical implications, the DL model overcomes the problem of non-repeatability with a genuine alternative. Despite decades of extensive and corroborating research, this alternative is still in its infancy and is only slowly beginning to understand the scope of its full effect on performance, brain, and metabolism [111]. Nevertheless, several elements of this model have already been imitated under different names, thereby indirectly validating its potential. By incorporating principles derived from physics, neurophysiology, pedagogy, and neural network research, bridges to other scientific disciplines are established, hence fostering more holistic collaborations in the future. However, these principles can only provide general guidance when studying a constantly changing biological system, underscoring the ongoing necessity to deepen our understanding of the individuality of changes over time as a fundamental characteristic of living beings [11,112].

## Data Availability

The data presented in this study are available in supplementary material here.

## Supplementary Materials

The following supporting information can be downloaded at: https://www.mdpi.com/article/10.3390/sports12010005/s1, Supplementary file S1: Julius_Apidogo_BB-HB-VB_DL-CI 2023.xlsx.
